# Predicting correlated outcomes from molecular data

**DOI:** 10.1093/bioinformatics/btab576

**Published:** 2021-08-06

**Authors:** Armin Rauschenberger, Enrico Glaab

**Affiliations:** Luxembourg Centre for Systems Biomedicine (LCSB), University of Luxembourg, 4362 Esch-sur-Alzette, Luxembourg; Luxembourg Centre for Systems Biomedicine (LCSB), University of Luxembourg, 4362 Esch-sur-Alzette, Luxembourg

## Abstract

**Motivation:**

Multivariate (multi-target) regression has the potential to outperform univariate (single-target) regression at predicting correlated outcomes, which frequently occur in biomedical and clinical research. Here we implement multivariate lasso and ridge regression using stacked generalization.

**Results:**

Our flexible approach leads to predictive and interpretable models in high-dimensional settings, with a single estimate for each input–output effect. In the simulation, we compare the predictive performance of several state-of-the-art methods for multivariate regression. In the application, we use clinical and genomic data to predict multiple motor and non-motor symptoms in Parkinson’s disease patients. We conclude that stacked multivariate regression, with our adaptations, is a competitive method for predicting correlated outcomes.

**Availability and implementation:**

The R package joinet is available on GitHub (https://github.com/rauschenberger/joinet) and cran (https://cran.r-project.org/package=joinet).

**Supplementary information:**

Supplementary data are available at *Bioinformatics* online.

## 1 Introduction

For clinical diagnosis and prognosis, multinomial (multiclass) classification is often more relevant than binary classification (Biesheuvel *et al.*, 2008; de Jong *et al.*, 2019), because it exploits and provides more information. Similarly, multivariate (multi-target) as compared to univariate (single-target) classification or prediction might often be more clinically relevant. Classifying patients into diseases that are not mutually exclusive, for example, requires a multivariate approach (cf. Vega, 2021). Teixeira-Pinto *et al.* (2009) explain why many applications involve multiple outputs rather than a single output: ‘lack of consensus on the most important [output]’, ‘desire to demonstrate effectiveness on [multiple outputs]’ and ‘disease complexity is often [better characterized by multiple outputs]’. Recent applications with multiple outputs include predicting mental illness and criminal behaviour of soldiers (Rosellini *et al.*, 2017), predicting various conditions of anaesthesia patients (Wang *et al.*, 2019) and predicting clinical outcomes after severe injury (Christie *et al.*, 2019). Although multiple outputs are commonly available, they are not commonly used for predictive modelling.

In a prediction problem with multiple outputs, which may represent different symptoms of the same disease, we could fit one *univariate* regression for each output, or one *multivariate* regression for all outputs. Exploiting the correlation among outputs, multivariate regression potentially improves the prediction of the output(s) of interest. Waegeman *et al.* (2019) describes the use of stacked generalization (also known as ‘stacking’) for multivariate regression (Wolpert, 1992; Breiman and Friedman, 1997). Here we adapt this approach to ridge and lasso regression, which are generalized by elastic net regression (Zou and Hastie, 2005), in order to estimate interpretable and predictive models in high-dimensional settings. As an implementation, we provide the package joinet for the R statistical computing environment.

Stacked multivariate regression involves multiple univariate regressions in two layers. In the base layer, we regress each output on all inputs, and in the meta layer, we regress each output on all cross-validated linear predictors from the base layer. Since combining linear predictors is equivalent to combining estimated coefficients, we construct a single estimate for each input–output effect (Rauschenberger *et al.*, 2021). Compared to multiple univariate regressions, stacked multivariate regression increases the predictive performance, while maintaining model interpretability. Thus, the proposed approach shares the benefits of ensemble learning methods in terms of predictivity without their usual limitations in terms of providing uninterpretable ‘black box’ models.

Friedman *et al.* (2010) have implemented elastic net regression (Zou and Hastie, 2005) for many univariate families and the multivariate Gaussian family (R package glmnet). We extend this implementation to multivariate outputs from the Gaussian, binomial and Poisson families through stacked generalization. Alternative multivariate predictive methods include multivariate adaptive regression splines (Friedman, 1991, R package earth), sparse partial least squares (Chung and Keles, 2010, R package spls), multivariate regression with covariance estimation (Rothman *et al.*, 2010, R package MRCE), regularized multivariate regression for identifying master predictors (Peng *et al.*, 2010, R package remMap), multivariate random forest (Segal and Xiao, 2011, R package MultivariateRandomForest), signal extraction for sparse multivariate regression (Luo and Qi, 2017, R package SiER), multivariate cluster elastic net (Price and Sherwood, 2017, R package mcen), Gaussian process modelling (Bostanabad *et al.*, 2018, R package GPM) and regularized multi-task learning (Cao *et al.*, 2019, R package RMTL). Furthermore, Xing *et al.* (2020) also implemented multi-task prediction using stacking (R package MTPS), but their approach does not meet our objective to increase the predictive performance without decreasing model interpretability (see Section 5).

There are different prediction types for multivariate regression. When modelling two binary events, for example, we might want to predict the marginal probability of an event, i.e. $P(Y_{1}=1)$ or $P(Y_{2}=1)$, the joint probability of both events, i.e. $P(Y_{1}=1\cap Y_{2}=1)$, or the conditional probability of one event given the other event, i.e. $P(Y_{1}=1|Y_{2}=1)$ or $P(Y_{2}=1|Y_{1}=1)$. Wilkinson *et al.* (2021) illustrate marginal, joint and conditional (marginal or joint) prediction in the context of species distribution modelling. We focus on *marginal* prediction, i.e. exploiting the correlation between outputs to improve the prediction of each output separately. For modelling multiple binary outputs, however, *joint* prediction might be more relevant (Dudbridge, 2020), i.e. modelling the correlation between events to predict the simultaneous occurrence of multiple events. See Martin *et al.* (2021) for a comparison of approaches to marginal and joint prediction of multiple binary outputs.

## 2 Materials and methods

Let the *n *×* p* matrix ***X*** denote the inputs (e.g. clinical or molecular data), and let the *n *×* q* matrix ***Y*** denote the outputs (e.g. multiple clinical measures), where *n* is the sample size, *p* is the number of inputs and *q* is the number of outputs. We will use the inputs (independent variables) to predict the outputs (dependent variables). Let *i* in {1,…,n} index the samples, *j* in {1,…,p} index the inputs, and *k* and* l* in {1,…,q} index the outputs. For sample *i*, the entries *X_ij _*and* Y_ik_* represent input *j* and output *k*, respectively. We allow for high-dimensional settings (p≫n) and for outputs generated from different univariate distributions (Gaussian, binomial, Poisson).

In the base layer, we regress each output on all inputs ***X***. For any sample *i* and output *k*, the base model equals

$$E[Y_{ik}]=h_{k}^{-1}\left(\beta_{0k}+\sum_{j=1}^{p}\beta_{jk}X_{ij}\right)\;,$$

where $h_{k}(\cdot )$ is the link function (identity, logit, log), $\beta_{0k}$ is the unknown intercept and $\beta_{k}={\left(\beta_{1k},\ldots ,\beta_{pk}\right)}^{⊺}$ are the unknown slopes. The slope $\beta_{jk}$ represents the effect of input *j* on the linear predictor for output *k*. We estimate the *q* base models by maximizing the penalized likelihoods, under lasso $\left(L_{1}\right)$ or ridge $\left(L_{2}\right)$ regularization (Zou and Hastie, 2005), which render sparse or dense models, respectively.

For each output, we tune the regularization parameter by *k*-fold cross-validation. Let the *n *×* q* matrix ${\hat{H}}^{\left(\text{cv}\right)}$ represent the cross-validated linear predictors (‘out-of-fold’), where ${\hat{H}}_{ik}^{\left(\text{cv}\right)}$ is the entry for sample *i* and output *k*:

$${\hat{H}}_{ik}^{\left(\text{cv}\right)}={\hat{\beta }}_{0k}^{-\kappa (i)}+\sum_{j=1}^{p}{\hat{\beta }}_{jk}^{-\kappa (i)}X_{ij}\;,$$

where the superscript −κ(i) indicates that the regression coefficients are estimated without using the fold including sample *i*. Using *cross-validated* rather than *fitted* linear predictors reduces leakage of information from the outputs to the inputs for the meta models (level-one data).

In the meta layer, we regress each output on all linear predictors ${\hat{H}}^{\left(\text{cv}\right)}$. For any sample *i* and output *k*, the meta model equals

$$E[Y_{ik}]=h_{k}^{-1}\left(\omega_{0k}+\sum_{l=1}^{q}\omega_{lk}{\hat{H}}_{il}^{\left(\text{cv}\right)}\right)\;,$$

where $\omega_{0k}$ is the unknown intercept and $\omega_{k}={\left(\omega_{1k},\ldots ,\omega_{qk}\right)}^{⊺}$ are the unknown slopes. The slope $\omega_{lk}$ represents the effect of the cross-validated linear predictor for output *l* from the base model on the linear predictor for output *k* in the meta model. We estimate the *q* meta models under lasso $\left(L_{1}\right)$ regularization to avoid overfitting.

In many applications, it is reasonable to assume that all pairwise combinations of outputs (e.g. different measures for disease severity) are *positively* correlated, i.e. $\rho_{kl}>0$ for all *k* and* l* in {1,…,q}, potentially after additive inverse transformations of some outputs, i.e. $Y_{\cdot k}\to -Y_{\cdot k}$ for some *k* in {1,…,q}. We then impose non-negativity constraints on the slopes of the meta models, i.e. ${\hat{\omega }}_{lk}\geq 0$ for all *k* and* l* in {1,…,q}. Non-negativity constraints have proven useful in the case of strongly positively correlated predictors according to extensive simulation studies (Breiman, 1996).

**Fig. 1.**
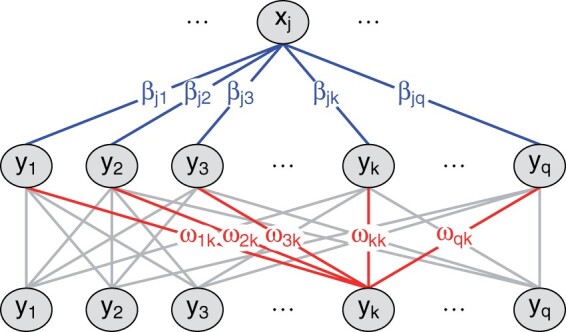
To estimate the effect of input *j* on the linear predictor for output *k*, we first estimate the effects of input *j* on the linear predictor for each output (base layer) and then estimate the effects of all cross-validated linear predictors on the linear predictor for output *k* (meta layer)

Given the estimated coefficients, we typically want to predict the outputs for previously unseen samples. The linear predictors of the meta learners combine the linear predictors of the base learners. For sample *i* and output *k*, the linear predictor equals

$$\begin{array}{c} \eta_{ik}^{⋆}={\hat{\omega }}_{0k}+\sum_{l=1}^{q}{\hat{\omega }}_{lk}{\hat{H}}_{il}={\hat{\omega }}_{0k}+\sum_{l=1}^{q}{\hat{\omega }}_{lk}\left({\hat{\beta }}_{0l}+\sum_{j=1}^{p}{\hat{\beta }}_{jl}X_{ij}\right)\\ ={\hat{\beta }}_{0k}^{⋆}+{\sum_{j=1}^{p}\hat{\beta }}_{jk}^{⋆}X_{ij}\;, \end{array}$$

where ${\hat{\beta }}_{0k}^{⋆}={\hat{\omega }}_{0k}+\sum_{l=1}^{q}{\hat{\omega }}_{lk}{\hat{\beta }}_{0l}$ and ${\hat{\beta }}_{jk}^{⋆}=\sum_{l=1}^{q}{\hat{\omega }}_{lk}{\hat{\beta }}_{jl}$. Hence, ${\hat{\beta }}_{jk}$ and ${\hat{\beta }}_{jk}^{⋆}$ are the initial and final estimated effects of input *j* on the linear predictor for output *k*, respectively, meaning that the final models have the same intuitive interpretation as the initial models in terms of input–output effects. For each input, stacking exchanges information among the estimated effects on the outputs, such that the final estimated effect on *one* output linearly combines the initial estimated effects on *all* outputs (Fig. 1).

Next, we consider two extensions to make stacked multivariate regression more generally applicable.

The first extension concerns *input–output* relationships: In some applications, we might want to exploit different inputs for modelling different outputs. For example, one group of inputs might be relevant for all outputs, but another group of inputs might only be relevant for some outputs. Let the *p *×* q* matrix ***W*** indicate which inputs (rows) are relevant for which outputs (columns). Specifically, let the entry in the j*th* row and the *k*th column indicate whether the *j*th input may be used for modelling the *k*th output in the base layer, with *W_jk_* = 0 meaning ‘no’ and *W_jk_* = 1 meaning ‘yes’. If an input may not be used for modelling an output (*W_jk_* = 0), the corresponding coefficient in the base model is set to zero (${\hat{\beta }}_{jk}=0$). In the meta layer, however, each output is regressed on all cross-validated linear predictors from the base layer. This means that even if the univariate prediction for output *k* may not depend on input *j* (base model), the multivariate prediction for output *k* might still depend on input *j* (meta model).

The second extension concerns *output–output* relationships: If all outputs are positively correlated, non-negativity constraints can help to introduce stability (see above). If some outputs are negatively correlated, however, we need to choose between not using any constraints and using non-negativity and non-positivity constraints. Let the *q *×* q* matrix ***V*** represent these contraints, where the entry in the *l*th row and the *k*th column indicates how the *l*th output may be used for modelling the *k*th output, with $V_{lk}=-1$ meaning ‘non-positive effect’, *V_lk_* = 0 meaning ‘no effect’, *V_lk_* = 1 meaning ‘non-negative effect’ and a missing value meaning ‘any effect’. The resulting constraints are ${\hat{\omega }}_{lk}\leq 0$ for a non-positive effect, ${\hat{\omega }}_{lk}=0$ for no effect and ${\hat{\omega }}_{lk}\geq 0$ for a non-negative effect. While the diagonal elements of the matrix ***V*** equal one, the off-diagonal elements may represent known or estimated relationships between outputs. Tentatively, we could check whether the Spearman correlation coefficient (between outputs *l* and* k*) is significantly negative ($V_{lk}=-1$), insignificant (*V_lk_* = 0) or significantly positive (*V_lk_* = 1) at the 5% level.

## 3 Simulation

We report the simulation study using the ademp framework (Morris *et al.*, 2019):

• **Aims:** In this simulation study, we compare the (marginal) predictive performance of different approaches to multivariate regression.
• **Data-generating mechanisms:** We repeatedly simulate data for *n* samples, *p* inputs, and *q* outputs, namely the *n* × *p* matrix ***X*** (inputs), the *p* × *q* matrix ***B*** (effects) and the *n* × *q* matrix ***Y*** (outputs), with a fixed random seed for reproducibility.
  For the *inputs*, we simulate the *n *×* p* matrix ***X*** from a multivariate Gaussian distribution with the constant mean 0 and the constant correlation *ρ_x_*, where $0\leq \rho_{x}\leq 1$. For the *effects*, we simulate the *p *×* q* matrix ***B*** from a multivariate Gaussian distribution with the constant mean 0 and the constant correlation *ρ_b_*, where $0\leq \rho_{b}\leq 1$. In each column, we leave the *m* largest values unchanged, and set the *p−m* smallest values equal to 0, where 0<m<p. Then the entry in row *j* and column *k* of ***B*** indicates whether input *j* affects output *k*, where *j* in {1,…,p} and *k* in {1,…,q}. For the *outputs*, we calculate the *n *×* q* linear predictor matrix H=XB, column-standardize ***H*** and simulate the *n *×* q* error matrix ***E*** from a standard Gaussian distribution. We then obtain the *n *×* q* matrix $Y=\sqrt{0.8}H+\sqrt{0.2}E$.
  We considered low-dimensional settings, sparse high-dimensional settings and dense high-dimensional settings. While the low-dimensional settings involve *p *=* *10 inputs with *m *=* *5 effects on each output, the sparse and dense high-dimensional settings involve *p *=* *500 inputs with *m *=* *10 or *m *=* *100 effects on each output, respectively. All settings involve n=10100 samples and *q *=* *3 outputs. We also varied the correlation between inputs and the correlation between effects. The correlation between inputs *ρ_x_* takes values in {0.0,0.1,0.3}, and the correlation between effects *ρ_b_* takes values in {0.0,0.5,0.9}. This leads to 3×3×3=27 settings in total, with various degrees of correlation between outputs.
• **Estimands/targets:** We trained and validated the models with $n_{0}=100$ samples, and tested the models with $n_{1}=10\;000$ samples, with 10 repetitions for each setting. This means that models are tested on previously unseen samples (‘holdout’). In each of the 270 iterations (27 settings times 10 repetitions), let $X_{0}$ and $Y_{0}$ denote the training data, and let $X_{1}$ and $Y_{1}$ denote the testing data. For each model, we compare its predicted outputs ($\hat{Y_{1}}$) with the true outputs ($Y_{1}$).
• **Methods:** We compared the proposed method (joinet) with one univariate method (glmnet) and eleven multivariate methods (glmnet, earth, spls, MRCE, remMap, MultivariateRandomForest, SiER, mcen, GPM, RMTL, MTPS).
  For standard univariate and multivariate regression (glmnet) and the base learners of stacked multivariate regression (joinet, MTPS), we used lasso regularization in the low-dimensional and the sparse high-dimensional settings, and ridge regularization in the dense high-dimensional settings.
 We aimed at comparable hyperparameter optimization, but this is too computationally expensive for three methods (MultivariateRandomForest, SiER, mcen). For internal cross-validation, we used the same 10 folds (glmnet, joinet, mcen), other 10 folds (spls, MRCE, MTPS, remMap, RMTL), because the implementations let the user choose the number of folds but not the fold identifiers, or 3 folds (SiER) due to the computational expense. We performed grid searches, specifically for glmnet and joinet: data-dependent sequence of 100 regularization parameters; spls: number of hidden components in {1,2,3,…,10} and thresholding parameter in {0.0,0.1,0.2,…,0.9} (i.e. 10 × 10); MRCE: both penalty parameters in $\left\{10^{1},10^{0.5},10^{0},\ldots ,10^{-4}\right\}$ (i.e. 11 × 11); remMap: both penalty parameters in $\left\{e^{0},e^{0.5},e^{1},\ldots ,e^{5}\right\}$ (i.e. 11 × 11); mcen: sequence of five penalty parameters, one possible cluster and cluster parameter in {0.1,1.1,2.1,…,5.1} (i.e. 5 × 6); and RMTL: both penalty parameters in $\left\{10^{1},10^{0.5},10^{0},\ldots ,10^{-4},0\right\}$ (i.e. 12 × 12). For MultivariateRandomForest, we used 100 trees, 5 features for each split and 5 samples for each node. For MTPS, we chose cross-validation residual stacking (Xing *et al.*, 2020), either ridge or lasso regression for the base learners (see above), and regression trees for the meta learner (Xing *et al.*, 2020), but a potential limitation is the ‘one-standard-error rule’ for the base learners (see Section 5).
• **Performance measures:** We measured the predictive performance based on the mean squared error of the testing samples, i.e. $\text{MSE}=\frac{1}{n_{1}\times q}\sum_{i=1}^{n_{1}}\sum_{k=1}^{q}{\left(Y_{1}{}_{ik}-{\hat{Y_{1}}}_{ik}\right)}^{2}$, but we divided the mean squared errors of all methods by the mean squared error of prediction by the mean (empty model, intercept-only model). These re-scaled mean squared errors are more comparable between different simulation settings, because 0% means that the predictions are perfect and 100% means that the predictions are as poor as those from prediction by the mean. For each method, we obtained 27 × 10 re-scaled mean squared errors (27 settings, 10 repetitions).
  For each setting and each repetition (27 × 10), we ranked the 12 multivariate methods by the re-scaled mean squared error. According to the mean rank, stacked multivariate regression (joinet) is among the top three most predictive methods in the low-dimensional settings (joinet: 2.1, glmnet: 2.8, GPM: 3.9), the sparse high-dimensional settings (joinet: 1.8, mcen: 3.0, spls: 3.4) and the dense high-dimensional settings (joinet: 2.1, spls: 2.6, RMTL: 3.2). For each of the 27 settings, we examined the 10 paired differences in re-scaled mean squared error between stacked multivariate regression and univariate regression. According to the one-sided Wilcoxon-signed rank test at the 5% level, stacked multivariate regression is significantly more predictive than univariate regression in 21 settings, namely in 6 low-dimensional, 8 sparse high-dimensional and 7 dense high-dimensional settings. As compared to the other multivariate methods, stacked multivariate regression outperforms univariate regression in more settings (joinet: 21, spls: 10, RMTL: 8, others: ≤6). Table 1 summarizes the re-scaled mean squared errors for each setting and each method (mean over 10 repetitions). We conclude that stacked multivariate regression leads to a competitive predictive performance.

**Table 1.** Mean loss of different models (re-scaled mean squared error, mean over 10 repetitions) in low-dimensional (top), sparse high-dimensional (centre) and dense high-dimensional (bottom) settings

*ρ_x_*	*ρ_b_*	*ρ_y_*	glmnet^a^	joinet	glmnet^b^	earth	spls	MRCE	remMap	MRF^c^	SiER	mcen	GPM	RMTL	MTPS
0.0	0.0	0.1	20.6	20.4	21.0	25.2	21.1	20.7	32.7	52.5	21.2	23.4	21.8	21.1	22.7
0.1	0.0	0.6	21.0	21.1	20.9	25.4	21.1	21.7	33.9	41.3	21.6	22.9	20.9	21.5	22.7
0.3	0.0	0.5	21.7	21.6	21.7	24.2	21.8	21.9	27.2	38.4	21.9	22.3	21.7	22.0	24.1
0.0	0.5	0.4	21.6	21.3	21.6	24.0	21.8	22.0	41.5	44.1	21.5	22.9	21.6	21.5	23.3
0.1	0.5	0.2	21.6	21.7	21.8	28.2	21.5	21.5	23.6	47.7	22.2	23.7	21.9	22.0	23.3
0.3	0.5	0.6	21.0	21.0	21.3	25.4	22.4	20.5	27.9	33.6	21.9	21.1	21.5	21.9	21.4
0.0	0.9	0.8	20.9	20.7	20.7	21.6	21.2	21.7	23.9	41.1	21.6	23.1	20.6	20.7	21.4
0.1	0.9	0.8	20.8	20.6	20.6	23.4	21.0	21.6	23.6	37.4	21.5	22.9	20.6	20.7	22.1
0.3	0.9	0.8	20.7	20.4	20.4	22.3	21.0	21.5	23.2	32.3	20.5	21.2	20.6	20.9	22.1

0.0	0.0	0.0	24.7	22.9	29.1	49.2	26.5	100.0	41.5	98.2	31.4	27.2	100.0	30.1	29.6
0.1	0.0	0.2	26.5	25.5	29.0	35.4	21.8	100.0	37.6	84.0	38.8	26.5	100.0	67.0	30.2
0.3	0.0	0.5	26.6	26.2	28.2	32.5	37.9	100.0	49.8	57.7	36.6	25.6	100.0	46.8	29.1
0.0	0.5	0.0	28.4	23.6	30.6	48.8	23.0	100.0	47.8	97.3	27.2	30.1	100.0	32.2	34.0
0.1	0.5	0.2	26.2	24.8	29.6	39.6	34.6	100.0	42.4	84.7	45.5	26.9	100.0	65.5	29.4
0.3	0.5	0.5	26.5	26.4	30.7	42.3	39.4	100.0	33.3	59.4	47.6	27.6	100.0	42.0	30.3
0.0	0.9	0.3	27.5	24.9	28.3	26.8	23.8	100.0	41.7	97.5	32.7	28.8	100.0	28.2	32.3
0.1	0.9	0.5	26.3	25.4	27.8	27.7	23.9	100.0	35.1	83.1	32.0	27.8	100.0	28.9	30.0
0.3	0.9	0.6	25.9	26.5	26.5	31.4	33.8	100.0	36.5	57.2	34.7	26.4	100.0	30.7	28.4

0.0	0.0	0.1	89.2	89.7	89.4	143.3	89.5	100.0	100.0	99.1	94.8	97.4	100.0	86.9	89.5
0.1	0.0	0.7	27.5	25.9	28.4	80.5	27.8	100.0	42.6	61.1	29.4	35.0	100.0	27.3	27.8
0.3	0.0	0.8	22.3	22.0	22.3	50.4	21.8	100.0	42.0	37.6	23.2	25.2	100.0	23.1	22.3
0.0	0.5	0.4	89.1	91.5	89.5	165.8	88.9	100.0	100.0	99.5	92.6	96.1	100.0	90.0	99.8
0.1	0.5	0.8	28.4	26.6	29.5	73.4	27.0	100.0	64.1	61.7	28.0	33.8	100.0	28.1	28.0
0.3	0.5	0.8	21.8	21.8	21.9	51.3	21.6	100.0	58.4	37.4	23.3	24.7	100.0	22.3	23.4
0.0	0.9	0.7	90.7	89.9	91.4	146.3	91.8	100.0	100.0	99.3	90.2	99.5	100.0	92.3	96.8
0.1	0.9	0.8	28.6	26.5	29.7	73.6	26.8	100.0	58.0	62.1	27.8	33.0	100.0	27.7	30.1
0.3	0.9	0.8	22.7	22.2	22.8	47.8	22.8	100.0	45.2	38.3	23.1	25.6	100.0	22.2	22.5

*Note*: The first three columns indicate the correlation between inputs (*ρ_x_*), the correlation between effects (*ρ_b_*) and the resulting mean correlation between outputs (*ρ_y_*). The other columns show the predictive performance of a univariate method (glmnet^a^), the proposed multivariate method (joinet) and eleven other multivariate methods (glmnet^b^, earth, spls, MRCE, remMap, MRF^c^, SiER, mcen, GPM, RMTL, MTPS). For each setting (row), the colour black indicates which multivariate methods are more predictive than the univariate method (glmnet^a^), and the underline indicates the most predictive method, based on the sharp (not rounded) numbers. ^a^Univariate linear regression with glmnet. ^b^Multivariate linear regression with glmnet. ^c^MultivariateRandomForest.

Multivariate regression can be computationally expensive. As compared to glmnet and joinet, the mean computation time is shorter for earth, but about five times longer for MRCE, remMap and MTPS, about ten times longer for RMTL, spls and mcen and above twenty times longer for SiER, GPM and MultivariateRandomForest. The computational efficiency of glmnet and thereby joinet stems from regularization paths via coordinate descent (Friedman *et al.*, 2010).

Another advantage of stacked multivariate regression (joinet) is its flexibility. First, it accepts multivariate outcomes from different families. Some of the alternative methods allow for different families in separate models but not in the same model. The current implementations accept continuous multivariate outcomes (glmnet, spls, MRCE, remMap, MultivariateRandomForest, SiER, GPM), either continuous or binary multivariate outcomes (earth, mcen, RMTL), or continuous and binary multivariate outcomes (joinet, MTPS). Second, it accepts missing values in the outcomes. An alternative would be to impute them by chained equations (van Buuren and Groothuis-Oudshoorn, 2011, R package mice) for the training data.

## 4 Application

We illustrate the application and assess the performance of stacked multivariate regression by analysing data from a clinical cohort study on Parkinson’s disease, which is part of the Parkinson’s Progression Markers Initiative (ppmi, Marek *et al.*, 2011). From clinical or genomic variables measured at baseline, we predict motor and non-motor symptoms measured at three follow-up visits. Supplementary Table A includes details on the pre-processing of the clinical and the genomic data.

The outputs to predict are the (total) scores from the following clinical assessment tools: Montreal Cognitive Assessment (moca, adjusted for education), Questionnaire for Impulsive-Compulsive Disorders in Parkinson’s Disease (quip), Movement Disorder Society Unified Parkinson’s Disease Rating Scale (mds-updrs, ‘off’), Geriatric Depression Scale (gds), Scales for Outcomes in Parkinson’s Disease – Autonomic Dysfunction (scopa-aut), Epworth Sleepiness Scale (ess), Benton Judgement of Line Orientation Test (bjlot) and Rapid Eye Movement (rem) Sleep Behaviour Disorder Questionnaire. We take the additive inverse transform of the moca and bjlot scores (y→−y) to ensure that all minima indicate ‘no symptoms’ and all maxima indicate ‘severe symptoms’ (which should render all pairwise combinations of outputs positively correlated). The inputs consist of 138 clinical variables (dataset 1) or 17714  rna-seq gene expression variables reflecting measurements from whole-blood samples (dataset 2). We restrict the analysis to samples with both types of inputs (*n *=* *242). Our aim is to build the most predictive model for each clinical assessment tool (moca, quip, updrs, gds, scopa, ess, bjlot, rem) and each clinical follow-up visit (first, second, third), i.e. 8 × 3 = 24 prediction problems. (In the following, we use the short terms ‘tool’ and ‘visit’, respectively.) We consider three types of inputs (clinical, genomic, both) and two types of regularization (lasso, ridge), i.e. 3 × 2 = 6 modelling approaches. This leads to 24 × 6 = 144 univariate regression models.

**Fig. 2.**
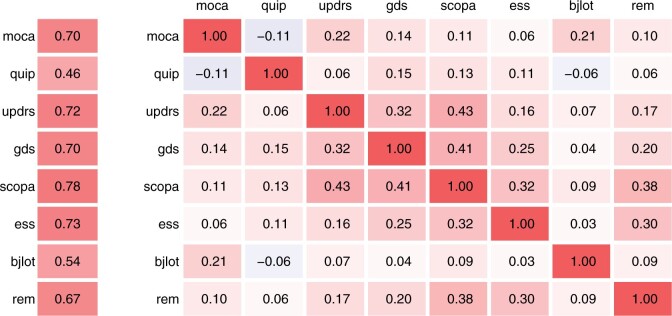
Spearman’s rank correlation coefficients. Left: correlation between outputs from the same tool at different visits (averaged across combinations of visits). Right: correlation between outputs from different tools at the same visit (averaged across visits)

Figure 2 summarizes the correlation between the outputs. Outputs from the same tool at different visits are strongly correlated (left), and outputs from different tools at the same visit are weakly correlated (right). The mean correlation between visits is strongest for scopa (Spearman’s ρ=0.78), and the mean correlation between tools is strongest for scopa and updrs (Spearman’s ρ=0.43). We suspect that, due to the correlated outputs, stacked multivariate regression has the potential to outperform univariate regression. In our two applications of multivariate regression, we share information among different clinical follow-up visits or between different clinical assessment tools, respectively, reflecting two common settings in clinical data analysis. In both cases, we first regress each output on all inputs, and then combine information from different outputs. In the first case, we support the prediction for one tool and one visit with the same tool at the other visits (‘support from other visits’), and in the second case, we support the prediction for one tool and one visit with another tool at the same visit (‘support from other tool’). Even if an output is not of interest itself, it can still support the prediction of other outputs, functioning as a ‘coaching variable’ (Tibshirani and Hinton, 1998).

We evaluate the predictive performance of univariate and multivariate regression by nested cross-validation, using *internal* cross-validation for hyperparameter optimization and *external* cross-validation for performance evaluation. While the holdout method would involve a single train-test split, external cross-validation involves multiple train-test splits. We first assign each sample to one external fold (out of five) and one internal fold (out of ten). In each external iteration (out of five), we train (parameter estimation) and validate (hyperparameter optimization) the models with four external folds (80%), and test (performance evaluation) the models with the other external fold (20%). The training and validation phase involves internal cross-validation. In each internal iteration (out of ten), we train the models with nine internal folds (80%×90%=72%) and keep the other internal fold (80%×10%=8%) for validation. After the last internal iteration in each external iteration, we tune the hyperparameters, and after the last external iteration, we evaluate the performance. This nested cross-validation scheme allows us to repeatedly use 80% of the samples for training and validation, and finally 100% of the samples for testing. We thereby test the methods on previously unseen data.

**Fig. 3.**
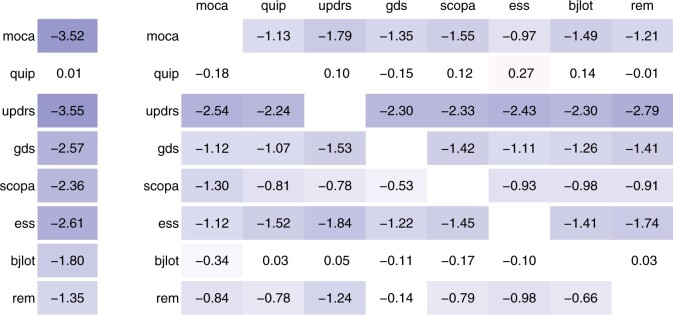
Percentage change in cross-validated mean squared error from univariate to multivariate regression. Left: we support the prediction for one tool at one visit with the same tool at other visits. Right: we support the prediction for one tool at one visit (row) with another tool at the same visit (column). All values are averaged across visits (1/2/3), regularization methods (lasso/ridge) and data types (clinical/omics/both), i.e. 18 settings

**Fig. 4.**
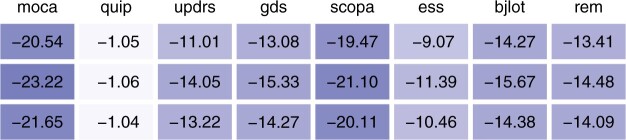
Percentage change in cross-validated mean squared error from prediction by the mean to univariate (first row) and multivariate regression (second row: support from other visits; third row: support from other tool). All values are averaged across visits (1/2/3), regularization methods (lasso/ridge), data types (clinical/omics/both) and coaching variables (third row only), i.e. 18 settings (first and second row) or 18 × 7 = 126 settings (third row)

Supplementary Figures A and B show the percentage change in cross-validated mean squared error from univariate to multivariate regression. The loss tends to decrease more strongly for the second visit than for the first and third visits, more under lasso than ridge regularization and more with combined clinical and genomic data than either clinical or genomic data. Figure 3 shows the mean percentage change in mean squared error. In this application, jointly modelling different visits (left) is more beneficial than jointly modelling different tools (right). It is most beneficial to support the prediction of the moca score with the moca scores at the other visits (left), or to support the prediction of the updrs score with the moca score at the same visit (right). We also assessed the predictive performance relative to prediction by the mean. Figure 4 shows the percentage change in cross-validated mean squared error from prediction by the mean to prediction by univariate and multivariate regression. The improvement is best for moca (above 20%) and worst for quip (about 1%). We observe the improvement tends to be larger for multivariate regression than for univariate regression. Overall, these empirical analyses show that stacked multivariate regression can be an effective means to improve the predictive performance as compared to univariate regression, if suitable correlated outcome data is available.

## 5 Discussion

Multivariate outputs frequently occur in biomedical and clinical research, because many symptoms and impairments associated with a complex disease cannot be captured by a single number. There are different sources of multivariate outputs: measurements of multiple attributes (e.g. different symptoms), multiple measurements of the same attribute (e.g. repeated measures) or multiple transformations of the same measurement (e.g. identity and logarithm).

If the outputs are neither too weakly nor too strongly correlated, we expect stacked multivariate regression to be more predictive than univariate regression. As the strength of the signal also matters, we cannot provide any thresholds for the correlation. If the inputs predict one output very badly, this output cannot provide support to the other outputs. And if the inputs predict one output very well, this output does not require support from the other outputs. To find out whether multivariate regression outperforms univariate regression in a specific application, we propose to use the holdout method or cross-validation.

Although the proposed multivariate model combines predictions from multiple univariate models, it has comparable interpretability to a univariate model, because stacking linear predictors is equivalent to pooling regression coefficients. The proposed method therefore shares the usual advantage of ensemble learning (high predictivity) but not the usual disadvantage (low interpretability). Though another approach to multi-target prediction (Xing *et al.*, 2020) can also combine two layers of penalized regression, our approach not only provides (i) a coefficient matrix that links the inputs to the *univariate* predictions and (ii) a coefficient matrix that links the univariate to the multivariate predictions but also a (iii) coefficient matrix that directly links the inputs to the *multivariate* predictions. This facilitates the biological interpretation of the statistical model. If non-linear effects might be important and if the aim is to maximize predictivity regardless of interpretability, however, we recommend the approach from Xing *et al.* (2020) with regression trees, quadratic discriminant analysis or *k*-nearest neighbour classification, because the proposed method only estimates linear effects.

The one-standard-error rule normally renders penalized regression models more parsimonious but not significantly less predictive. We argue, however, that the one-standard-error rule should not be used in stacked multivariate regression. Although it normally does not make models significantly less predictive, it still makes them less predictive (unless there is overfitting). Whereas it affects each output only once in univariate regression, it affects each output multiple times in stacked multivariate regression (once in the meta learner and once in each included base learner). And multiple insignificant decreases in predictivity can sum up to a significant decrease. Therefore, in stacked multivariate regression, the one-standard-error rule might lead to significantly worse predictions.

It would be interesting to extend stacked multivariate regression to settings with not only many inputs but also many outputs. This would for example be relevant for predicting gene expression values from other molecular data. Lutz and Bühlmann (2006) showed that multivariate modelling can outperform univariate modelling even if the number of outputs is high-dimensional. Our approach, however, involves cross-validating two regression models for each output, which would be too computationally expensive in this case.

The proposed method is of special interest for biomedical research because multiple outputs and high-dimensional inputs are becoming the rule rather than the exception in this domain. Our flexible approach allows for outputs with missing values, output-specific probability distributions and output-specific loss functions. It provides a general framework for prediction problems with multiple outputs and high-dimensional inputs. The R package joinet is available on GitHub (https://github.com/rauschenberger/joinet) and cran (https://cran.r-project.org/package=joinet).

**Key points**

- Biomedical prediction problems often include many inputs (e.g. molecular data) and multiple outputs (e.g. clinical data).
- Multivariate regression (‘multitasking’) outperforms univariate regression (‘single-tasking’) at predicting correlated outputs.
- Stacked multivariate regression leads to predictive and interpretable models in high-dimensional settings.

## Supplementary Material

btab576_Supplementary_Data
