# Supplementary material for: Predicting correlated outcomes from molecular data
Source: Bioinformatics. 2021 Aug 6;37(21):3889–95. doi: 10.1093/bioinformatics/btab576 (PMC10186156; doi:10.1093/bioinformatics/btab576)
Supplement: btab576_Supplementary_Data [file btab576_supplementary_data.pdf]

Supplementary material for “Predicting correlated outcomes from molecular data”

Armin Rauschenberger, Enrico Glaab

Luxembourg Centre for Systems Biomedicine (LCSB),
University of Luxembourg, Esch-sur-Alzette, Luxembourg

This supplementary material includes further information on the application. Table A describes the pre-processing of clinical and genomic data. Figures A and B show the change in predictive performance from univariate to multivariate regression. For reproducibility of the simulation and the application, see the vignettes of the R package `joinet` (https://github.com/rauschenberger/joinet, https://cran.r-project.org/package=joinet).

Table A: Pre-processing of clinical and genomic data.

clinical inputs	From the curated baseline clinical data (“PPMI_Baseline_Data_02Jul2018.csv”), a table with 683 rows (samples) and 134 columns (variables), we retain the 423 samples with Parkinson’s disease (i.e. apprdx=1) and the 130 clinical variables (i.e. without “site”, “apprdx”, “event_id” and “symptom5_comment”). After imputing the missing values ($\approx 3\%$) by chained random forests (R package `missRanger`), we transform the categorical variables (i.e. “apoe”, “snca_rs356181”, “snca_rs3910105” and “mapt”) to dummy variables. This leads to a matrix with 423 rows (samples) and 138 columns (variables).
genomic inputs	We use the count matrix generated by Zhi Zhang’s RNA-Seq pipeline (Python package `sumrnaseq.main 0.1.0`, https://pypi.org/project/sumrnaseq.main/). From the baseline genomic data (“ppmi_rnaseq_bl_pd_hc-2019-01-11.Rdata”), a table with 363 rows (samples) and 57 820 columns (variables), we remove the transcripts without an HGNC gene symbol (i.e. names starting with ENSG0000 or ENSGR0000). This reduces the dimensionality by approximately 38%. After calculating the library sizes (total count for each sample) and the abundances (total count for each gene), we remove the genes with a low abundance (less than 10 times the sample size), and adjust the counts for different library sizes (R package `edgeR`). We then Anscombe-transform ($x \rightarrow 2\sqrt{x+3/8}$) and standardise the counts (mean zero and variance one for each gene). This leads to a matrix with 363 rows (samples) and 17 714 columns (variables).
clinical outputs	From the curated follow-up clinical data (“PPMI_Year_1-3_Data_02Jul2018.csv”), a table with 1 783 rows (samples, visits) and 124 columns (variables), we retain the visits of interest (i.e. “V04”, “V06” and “V08” but not “ST”) and the variables of interest (i.e. “moca”, “quip”, “updrs_totscore”, “gds”, “scopa”, “ess”, “bjlot” and “rem”) together with the sample and visit identifiers (“PATNO” and “EVENT_ID”). We then reshape the data from the long to the wide format. This leads to a matrix with 654 rows (samples) and $3 \times 8 = 24$ columns (variables, visits).

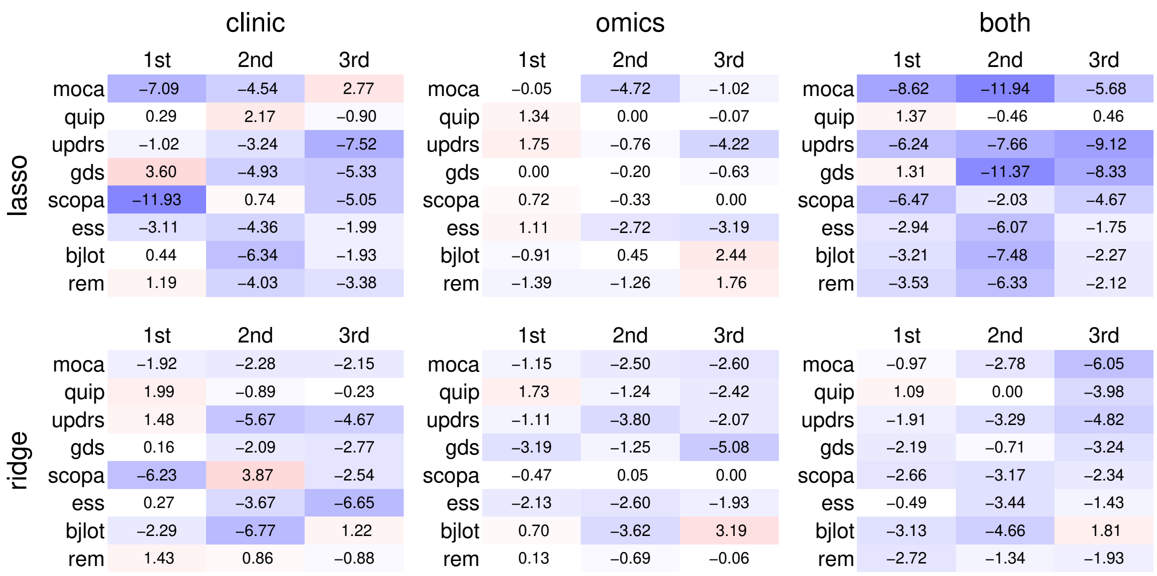

Figure A: Percentage change in cross-validated mean squared error from univariate to multivariate regression, with decreases in blue and increases in red. We consider six different settings, namely lasso or ridge regularisation (outer row) for clinical data, omics data, or both (outer column). Each multivariate model supports the prediction for one tool (inner row) at one visit (inner column) with the same tool at the other visits.

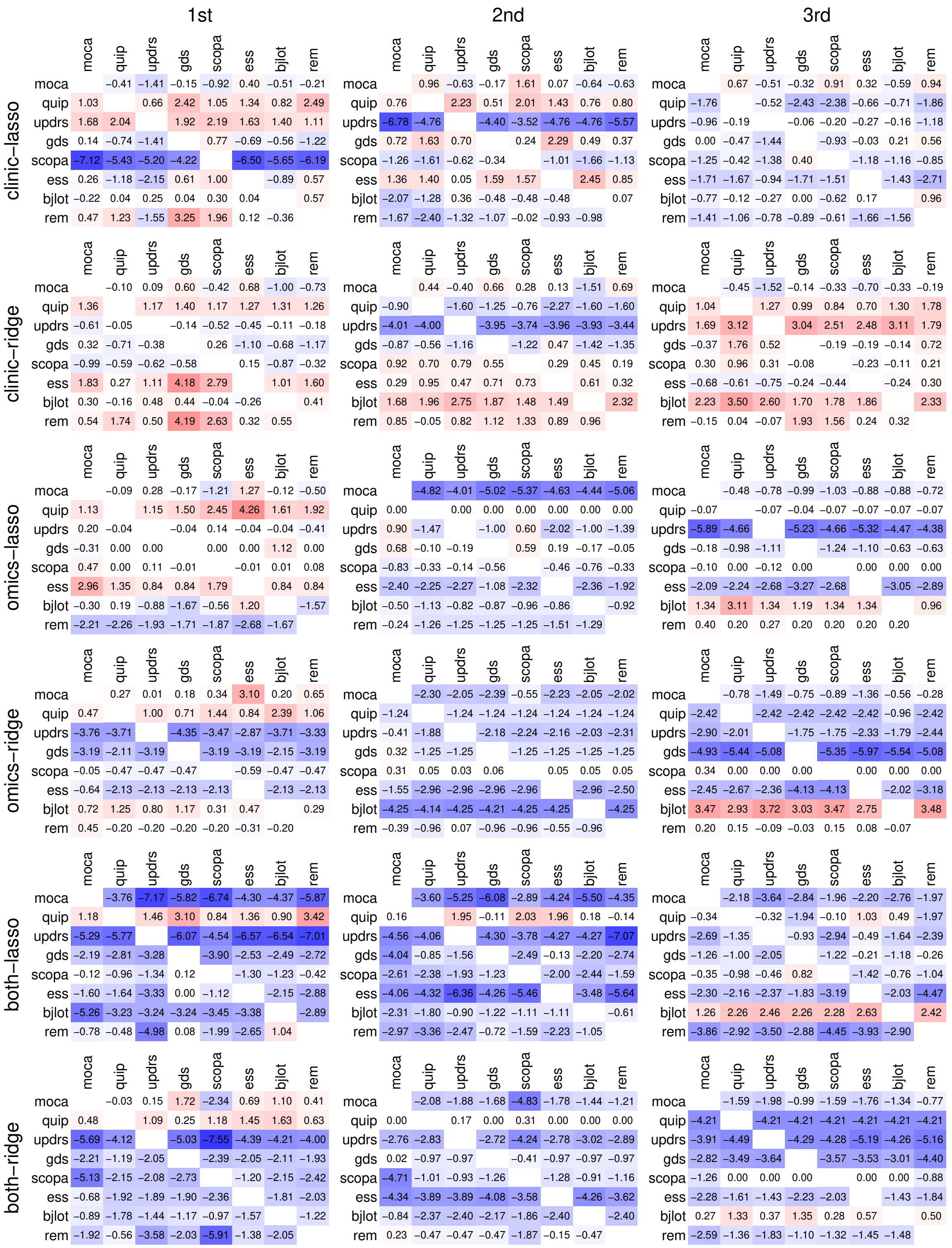

Figure B: Percentage change in cross-validated mean squared error from univariate to multivariate regression, with decreases in blue and increases in red. We consider six different settings, namely lasso or ridge regularisation for clinical data, omics data, or both (outer row), and analyse each visit separately (outer column). Each multivariate model supports the prediction for one tool at one visit (inner row) with another tool at the same visit (inner column).
